# The Association between Perceived Family Financial Stress and Adolescent Suicide Ideation: A Moderated Mediation Model

**DOI:** 10.3390/bs13110948

**Published:** 2023-11-17

**Authors:** Qi Yang, Wenyu Zhang, Huan Wu, Baozhen Huang, Chenyan Zhang, Gengfeng Niu

**Affiliations:** 1 School of Humanities, Tongji University, Shanghai 200092, China; 2 School of Marxism, Zhejiang Gongshang University, Hangzhou 310018, China; 3 Mental Health Education and Counselling Centre, Guangzhou College of Commerce, Guangzhou 511363, China; wdyhewh@163.com; 4 College of Education and Arts, Ningde Normal University, Ningde 352100, China; 5 Tongji Hospital Affiliated to Tongji Medical College of Huazhong University of Science & Technology, Wuhan 430030, China; zcymind@163.com; 6 Key Laboratory of Adolescent Cyberpsychology and Behavior (CCNU), Ministry of Education, Wuhan 430079, China; 7 Key Laboratory of Human Development and Mental Health of Hubei Province, School of Psychology, Central China Normal University, Wuhan 430079, China

**Keywords:** suicidal ideation, perceived family financial stress, parent–child attachment, depression, adolescents

## Abstract

Nowadays, suicide (especially adolescents’ suicide) has been an increasingly prominent social problem worldwide; suicide ideation, as an important predictor, has been the focus of relevant studies and practices. Against this background, the present study aimed to examine the association between perceived family financial stress and adolescents’ suicidal ideation, as well as the potential roles of depression and parent-child attachment. A sample of 526 junior middle school students was recruited voluntarily to participate in this cross-sectional study, and the results indicated that the prevalence of suicidal ideation among junior high school students was 15.45%; perceived family financial stress was positively associated with suicidal ideation, and depression could significantly mediate this relation; parent–child attachment significantly moderated the mediating effect of depression (in particular, the relation between depression and suicidal ideation); specifically, this relation was stronger among adolescents with lower values of parent–child attachment. These findings could deepen our understanding of the influences of perceived family financial condition and the risky factors of adolescents’ suicidal ideation, which could provide guidance for the prevention and intervention of adolescents’ depression and suicidal ideation.

## 1. Introduction

Adolescent suicide has become a major global public health problem around the world. Statistics indicate that, in the last decade, suicide has become the second leading cause of death among adolescents, with a nearly tripled suicide rate observed among individuals from 10- to 14-year-olds [1,2,3]. Adolescents’ suicide behavior may cause great and long-term harm at social, family, and individual levels, such as occupying medical recourses [4,5], causing psychological pain and burden to the family [6,7], and undermining the mental health of individuals who experience suicide bereavement [8]. Moreover, adolescence is a unique developmental stage characterized by significant physiological, emotional, and social changes. This adolescence renders adolescents encounter various stresses of social development [9]. So, adolescents are at high risk of suffering from health and adaptation problems, which would further increase the risk of suicidal behavior. To clearly examine suicidal behavior, many studies have focused mainly on suicidal ideation, given that suicidal ideation is not only an inevitable stage in the suicide formation process but also could serve as the sensitive predictor for suicidal behavior [10,11,12]. Based on these views, it is of great significance to identify the influencing factors and mechanism underlying suicidal ideation so as to promote the prevention and intervention in suicidal behavior in adolescents.

Many studies have examined the influencing factors of suicidal ideation and found that various factors, both environmental factors (e.g., family financial status and social connectedness [13,14]; Cyber-victimization [15]) and individual factors (e.g., attachment anxiety, depression [16]; gender, age, mental health [17]; personality dimensions, trait anxiety [18]), contributed to the development of suicidal ideation. Among these factors, family-related factors are the key factors greatly influencing adolescents’ various development and adaptation outcomes [19,20,21,22,23]. Regarding suicidal ideation, previous studies mainly focused on parenting-related issues (such as parenting practices and parental mental health) [22], and the objective family environment has been largely neglected (such as family financial status) [24], which should be examined to further uncover the relation between family and adolescents’ adaptation, and the risk factors of suicidal ideation in the family. Due to the negative impact of COVID-19 on the global economy and individuals’ mental health, examining this issue is of great significance in the current post-pandemic era.

Family or household income in adolescence is consistently and inversely associated with suicide risk [25], and it has been well established that there exists a significant relation between perceived family financial difficulties and suicidal ideation, especially among adolescents. For example, Fiksenbaum [13] found that perceived economic hardship was positively related to suicidal ideation among various populations, including adolescents [26,27], and in different cultures [11,26,27]. Moreover, many studies have shown that individuals with poor socioeconomic status are more likely to suffer from suicidal ideation and suicidal behaviors [28,29]. Thus, it’s clear that perceived family financial stress was one important factor in predicting adolescent suicidal ideation. However, it remains unclear how these adolescents with higher family financial stress experience suicidal ideation. This study aimed to further examine the mechanism underlying this relation.

### 1.1. The Mediating Role of Depression

At the same time, poverty is closely linked to emotion-related problems [30,31,32]. In particular, perceived family financial stress was also found to be closely associated with depression among adolescents [33,34]. As family financial conditions reflect the potential resources that the family could provide to promote adolescents’ development and adaptation, family financial stress could negatively influence individuals directly and indirectly. Studies also have suggested that financial stress could exert a negative impact on parents, in turn, which leads to depression in adolescents [34]. In addition, these factors, such as housing conditions, household debt, and quality of life factors, which reflect family financial status, have been linked to depression [35,36,37].

Depression is an internalizing problem containing a wide range of symptoms, such as feelings of hopelessness, helplessness, emptiness, and worthlessness [38]. Adolescents are more likely to suffer from various emotional problems, including depression. According to the Blue Book on Mental Health in China 2022, the detection rate of depression among adolescents in China was 15–20% in 2022. These negative and painful feelings accompanying depression, which are considered high risks of suicidal ideation, tend to cause individuals to generate suicidal ideation and even commit suicide [39,40]. Moreover, relevant empirical evidence has been accumulated that depression would increase the risk of self-injury, attempted or completed suicide, and other risky behaviors [41,42,43,44,45]. Thus, perceived family financial stress may increase the risk of depression and further lead to suicidal ideation.

According to the Stimulus-Organism-Response model, organismic components (such as biological or psychological) may mediate the relations between external stimulation and personal outcomes [46]. In line with this model, individual perceptions of family financial stress and suicidal ideation could be separately considered stimulation and human thoughts. To be specific, family financial stress has a few direct influences on suicide ideation, but it can exert negative effects on suicide ideation through the proximate factors of suicide ideation, such as depression, given that depression was an important and prominent predictor of suicide ideation. Meanwhile, depression is also susceptible to family and environmental risk factors, such as family financial stress. Based on this rationale, we chose depression as the mediator between family financial stress and suicide ideation. Ultimately, perceived family financial stress can lead to negative emotions such as hopelessness and helplessness, which can increase the risk of depression, which in turn reduces individuals’ ability to cope with stress, making them more vulnerable to suicidal ideation. These findings have been demonstrated among undergraduates [47,48]. Compared with college students, adolescents are in a unique developmental period and have a high risk of suffering from health and adaptation problems [9]. The maximum peak incidence of suicidal behaviors and suicidal ideation is also observed during adolescence [49]. Thus, it was hypothesized that depression could mediate the relationship between perceived family financial stress and suicidal ideation.

### 1.2. The Moderating Role of Parent–Child Attachment

It’s true that not all individuals would be equally affected by the same environmental factors, and much attention has been attracted to the potential protective factors in the relation, which may have important implications for relevant intervention practices. Parent–child attachment involves the development of emotional connections with significant individuals. The process of establishing the parent–child attachment is beneficial to gradually develop different emotional, cognitive, and behavioral strategies in order to obtain or maintain an internal sense of personal security. For instance, when individuals are threatened by their surroundings, the good-quality attachment activates the mental representation of relationship partners who regularly provide care and protection. This mental representation creates a sense of safety and security, which helps them successfully address threats [50]. That is, the attachment leads to progressive emotional growth, culminating in an internal sense of personal security that allows for the exploration of self and others in a safe environment [51,52]. It is an important positive factor that promotes children and adolescents’ good development and adaptation [53]. Substantial evidence has accumulated on the positive influences of secure attachments on individuals’ positive social–emotional competence, cognitive functioning, and physical and mental health, whereas children with insecure attachments are more at risk for negative outcomes in these domains [54]. Note that when individuals do not develop a secure representation, it does not necessarily mean that children are not attached to their attachment figures. Besides the direct positive effects, the parent–child attachment could also buffer the negative impacts of risk factors on adolescents’ adaptation [55]. Regarding suicidal ideation, previous studies have shown that parent–child attachment could reduce suicidal ideation among young teenagers [56,57]. Adolescents with stronger family communication and support can better handle life changes [58], which could further alleviate the link between depression and suicidal ideation, while those with weaker family communication and support may have less chance of obtaining support from their parents, heightening the relationship between depression and suicidal ideation [59,60,61]. Additionally, parent–child attachment alleviates delinquency of ADHD adolescents and buffers the adverse effects of sibling bullying on adolescent mental health [57]. Hence, parent–child attachment serves as a buffer function and moderator in the relationship between depression and suicidal ideation among adolescents.

To sum up, this study aimed to examine the relationship among perceived family financial stress, depression, parent–child attachment, and suicidal ideation in Chinese adolescents. Drawing on the stress-psychopathology model, and attachment theory, the current study aimed to identify the internal mechanisms that underlie this relationship. By shedding light on the understanding of the underlying status and forming mechanisms of suicidal ideation among adolescents, this study is expected to provide valuable insights into the prevention and intervention of adolescent suicide.

## 2. Materials and Methods

### 2.1. Participants

A total of 526 adolescents from two junior high schools located in central China, were recruited to participate in this cross-sectional study voluntarily. They were administered to complete a pencil-paper questionnaire during their class. Then, 47 participants whose score on a lie subscale was more than 4 were excluded for further analysis. This exclusion criteria resulted in the final valid sample size of 479 students (219 females) with a mean age of 14.29 years (SD = 0.81). Each participant and their parents provided informed consent for participation in the study. The study was approved by the Ethics Committee of the corresponding author’s University.

### 2.2. Measurement

#### 2.2.1. Family Financial Stress

The family financial stress questionnaire was used to assess the frequency in the last 12 months the adolescent perceived and experienced family financial stress [61], with 4 items. The questionnaire uses five-point Likert-type response options (1 = “none of the time”, 5 = “all of the time”). Higher scores indicate greater levels of economic strain. In the present study, Cronbach’s alpha for this scale was 0.71.

#### 2.2.2. Parent–Child Attachment Relationship

The Chinese version of the parent and peer attachment (IPPA) inventory was employed to assess the level of the mother/father–child attachment relationship [62], which contains 20 items. The questionnaire adopts a five-point Likert-type response option (1 = “strongly disagree”, 5 = “strongly agree”). Higher scores indicate better quality of parent–child attachment. In the current study, Cronbach’s alpha for this scale was 0.86.

#### 2.2.3. Depression

The Chinese version of the depression self-rating scale for children was used to measure the level of depression [63], which contains 18 items. Two items were excluded as they were not in accordance with other items statistically. The questionnaire uses three-point Likert-type response options (0 = “hardly ever”, 1 = “some of the time”, 2 = “often”). Higher scores suggest more severe levels of depression. In the present study, Cronbach’s alpha for this scale was 0.73.

#### 2.2.4. Suicidal Ideation

The self-rating idea of the suicidal scale was adopted to assess suicidal ideation [64], which includes 26 items. This scale can be divided into four dimensions: despair, optimism, sleep, and lie. The questionnaire uses two-point response options (0 = “yes”, 1 = “no”). Participants who obtain scores higher than 4 on the lie subscale are considered invalid data. Higher scores suggest high levels of suicidal ideation. In the current study, Cronbach’s alpha for this scale was 0.74.

### 2.3. Common Method Bias Test

As all the data were collected through self-report, potential common method bias (CMB) may exist and threaten the reliability of the present study. Though procedural remedies in this study were employed by mature measure tools, using reverse-scored items, protecting respondent anonymity, and polygraph items, to guarantee the rigor of the results, Harman’s one-factor test was further adopted and conducted to test whether there exists a significant common method bias. Specifically, the study tests the hypotheses that a single factor emerges from the exploratory factor analysis or that one general factor accounts for the majority of the covariance among the measures. The statistical test result showed that the first factor only accounted for 16.64% of the total variance explained, less than the critical value of 40%, suggesting there was no significant CMB in this study.

## 3. Results

### 3.1. Descriptive Results and Correlational Analysis

Forty-seven subjects whose scores on the lie subscale were more than four were excluded, and these participants whose total scores on the other three subscales were higher than eleven were considered as having suicidal ideation, which results in a prevalence of 15.45%.

Table 1 presents means, standard deviations, and inter-correlations for all variables. An inspection of the partial correlations with gender and age as the control variables revealed that depression was positively related to perceived family financial stress (r = 0.18, *p* < 0.001) and suicidal ideation (r = 0.69, *p* < 0.001), whereas parent attachment relationship was inversely and separately related to depression (r = −0.50, *p* < 0.001) and suicidal ideation (r = −0.45, *p* < 0.001).

### 3.2. Tests of Moderated Mediation

Based on the theoretical hypotheses and correlational analyses mentioned above, the present study further tested the moderated mediation model between family financial stress, depression, parent–child attachment, and suicide ideation by the package PROCESS for SPSS software [65] (see Table 2). Model 14 (which fits well into the hypothesized model) was adopted to estimate a moderated mediation model with controlling gender and age. The results of the mediation analysis indicated that perceived family financial stress positively predicted depression (β = 0.07, SE = 0.02, t = 4.76, *p* < 0.001), while depression, in turn, positively predicted suicidal ideation with (β = 0.54, SE = 0.04, t = 15.66, *p* < 0.001). Furthermore, the cross-product term between depression and parent–child attachment on suicidal ideation was significant (β = −0.13, SE = 0.04, t = −3.07, *p* < 0.01), suggesting that parent attachment can moderate the impact of depression on suicidal ideation.

**Figure 1:**
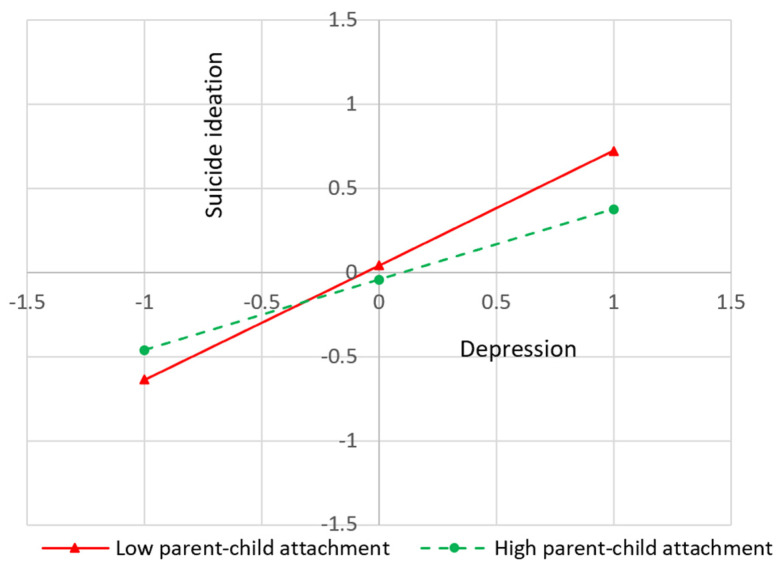
A moderating effect of parent–child attachment.

To examine the conditional indirect effect proposed, we conducted significance tests based on the hypothesis that the conditional indirect effect equals zero at certain levels of the moderator (mean ±1 SD)—in this case, the parent–child attachment relationship. The results indicated that the indirect relationship between depression and suicidal ideation was relatively weak for adolescents with high scores (+1SD) in parent attachment, whereas the indirect relationship was relatively strong for children with low scores (−1SD) in parent attachment (see Table 3 and Figure 1). Thus, the conditional indirect effect decreased with an increasing level of parent attachment.

## 4. Discussion

Considering the prevalence of suicide and the development and lived realities of adolescents, the primary aim of the present study was to examine the association between perceived family financial stress and suicidal ideation among adolescents, as well as the mediating role of depression and the moderating role of parent–child attachment in this association. The results found that perceived family financial stress could negatively predict adolescents’ suicidal ideation by increasing the risk of depression, and this effect was more prominent among adolescents with low values of parent–child attachment.

### 4.1. The Mediating Role of Depression

The present study revealed a significant and positive correlation between adolescents’ perception of family financial stress and suicidal ideation, which is consistent with previous studies [25] and theoretical frameworks [66]. Previous studies demonstrated that individuals living in poverty (facing great financial stress) are more likely to experience negative life events and accrue fewer protective benefits from their environments [67,68,69]. According to the stress-psychopathology model, observable life stressors, such as financial stressors and academic challenges, greatly influence individuals’ mental health and well-being, which further contribute to the development of suicidal ideation and even suicidal behavior. This association has also been observed in various age groups, including young adults [70], older adults [71,72], and different cultures, such as Western [26,27] and Eastern cultures [71]. According to Bronfenbrenner’s bioecological theory of human development [24], family financial status forms a crucial part of individuals’ material resources within their home environment, which affects their mental well-being and mental health. Family financial stress may predict or be accompanied by many negative family factors, and that their family could fail to provide necessary and enough support and resources promoting their development. Moreover, adolescents, being in a sensitive stage of development, tend to compare themselves with their peers to improve their self-image. This tendency may amplify the role of family financial stress in contributing to suicidal ideation among adolescents. Hence, this study further emphasizes and verifies the crucial role of the family environment in promoting adolescent mental health.

More importantly, when depression was added as a mediating factor in explaining the relationship between perceived family financial stress and adolescent suicidal ideation, the direct effect of perceived economic stress on suicidal ideation became weaker, indicating a significant mediating effect of depression. This pattern of results is also consistent with previous research findings and the stress-psychopathology model. As suggested by the stress-psychopathology model, life stress (in this study primarily referring to family financial stress) is an observed and predisposing factor for mental health. The Stimulus–Organism–Response model also pictures that stimulation (e.g., family financial stress) and human behavior (e.g., suicide) are linked by an organismic component, including biological and psychological components [46]. Specifically, perceived family financial stress may affect inner mental status and further lead to suicidal ideation and even suicidal behavior. Moreover, in the present study, depression could be a mediator of the relation between family financial stress and suicidal ideation, given that suicidal ideation was an important factor in predicting individual suicide. Additionally, family financial stress may limit access to resources and activities that can promote positive mental health, such as counseling services, medication, and recreational activities. This lack of access to mental healthcare resources may lead to increased levels of depression, which, in turn, exacerbates thoughts of suicide [66]. Additionally, adolescents in middle school, who are in the sensitive stage of peer interaction and social comparison and who are more easily affected by negative environmental factors and peer pressure, may be more likely to exhibit depression among those with higher family financial stress and in turn, those with depression may exhibit stronger levels of suicidal ideation [12,73]. The mediating role of depression may suggest that adolescents with higher family financial status are more prone to experiencing suicidal ideation through depression. This finding further emphasizes the importance of emotional issues in adolescents living under perceived family financial stress.

### 4.2. The Moderating Role of Parent–Child Attachment Relationship

This study also further examines the individual differences in the relations—parent–child attachment moderates the way of depression to suicidal ideation. The research findings indicate the associations between depression and suicidal ideation were weak among adolescents with higher-quality parent–child attachment when compared to those with poor-quality attachment. The parent–child attachment relationship serves as an internal protective resource, buffering the negative impact of depression on suicidal ideation. The findings are also consistent with previous studies [56,57] and parent–child attachment relationship theory [52]. Parent–child attachment can provide a sense of emotional support and security, which can help to mitigate the negative effects of depression. Adolescents who feel emotionally secure in their relationships with their parents may be less likely to experience the full impact of depression and more likely to receive social support and encouragement to seek help when needed [74]. Moreover, those with high-quality parent–child attachment could co-accompany with other beneficial factors (such as better social skills, high self-esteem, and the tendency to express feelings), which could together buffer the negative effects of depression on suicidal ideation [75,76]. Conversely, adolescents with poor parent–child attachment may experience greater emotional distress when being confronted with depression and may be less likely to seek help or receive social support. The resulting sense of isolation and helplessness may increase the risk of suicidal ideation [77]. Data support a significant association between insecure attachment and depressive symptoms in children [78]. Additionally, this view was supported by many studies demonstrating that family function and parental warmth exert a significant and positive impact on teenagers’ suicidal ideation [79]. This issue seems to be consistent with the empirical data obtained in this study since the attachment of adolescents to parents represents a protective factor against the deleterious effect of depression on the development of suicidal ideation. Overall, the study provides new insights into the complex mechanisms between perceived family financial stress and adolescents’ suicidal ideation and extends the suicide psychopathology model by incorporating the buffer model of the parent–child attachment relationship.

### 4.3. Implications and Limitations

The present study could shed light on the mechanisms that underlie suicidal ideation in adolescents. Specifically, the study developed a moderated mediation model to examine the mechanism underlying the relationship between family financial factors and adolescents’ suicidal ideation—the mediating role of depression and the moderating role of parent–child attachment. Moreover, the study also has some practical implications, especially in the current post-pandemic era, as the global economy and individuals’ mental health have been greatly affected by COVID-19. Firstly, special attention should be paid to adolescents with high financial stress and educate them to reasonably look at the family environment. Then, measures should be taken to alleviate adolescents’ negative emotions (especially depression) and guide them to cope with these emotions positively. Additionally, parents should be encouraged to participate actively in their adolescents’ lives with a democratic parenting style, as this can help foster secure attachments with parents [80,81].

However, it is important to consider the limitations of this study. Firstly, the data collected was based on self-reported measures, and the study design was cross-sectional, which limits the ability to draw causal conclusions. Future research is encouraged to employ longitudinal or experimental designs to better understand the relationships among these variables. Secondly, the study was conducted among Chinese students, and cultural differences may result in different outcomes. For instance, how Eastern and Western teens handle negative emotions differs in intensity and reasoning, with Western teens expressing more directly and Eastern teens being more indirect and reserved, focusing on contextual factors. Therefore, future research could compare the results across distinct cultures. Thirdly, perceived family financial stress may not be consistent with actual poverty, and comparing their impact could be a valuable avenue for further research. Finally, the study could consider other buffering and environmental/familial factors that may affect the relationship between financial stress and suicidal ideation [26,82].

## 5. Conclusions

This study examined the potential mediating role of depression in the relationship between family financial stress and adolescent suicidal ideation, as well as the moderating effect of parent–child attachment on the relationship between depression and suicidal ideation. The findings emphasize the significance of understanding the adolescent perception of parent–child attachment, particularly when those with high levels of perceived family financial stress encounter depression and suicidal ideation. Although suicidal ideation is a complex issue influenced by various factors, parent–child attachment has been identified as a significant protective factor. Therefore, interventions aimed at improving the financial status and enhancing the quality of parent–child attachment may be effective in preventing or reducing adolescent suicidal ideation. Further research is essential to identify the various family financial circumstances that may lead to suicidal ideation and to examine other buffering factors that contribute to adolescent mental health.

## Figures and Tables

**Table 1:** Descriptive statistics and study variable inter-correlations with age and gender as the control variables.

	M (SD)	1	2	3	4
1. Perceived family financial stress	1.36 (0.56)	-			
2. Suicidal ideation	0.32 (0.20)	0.17 ***	-		
3. Depression	0.79 (0.23)	0.18 ***	0.69 ***	-	
4. Parent–child attachment	3.62 (0.65)	−0.16 ***	−0.45 ***	−0.50 ***	-

Note: M = Mean; SD = Standard Deviation. *** *p* < 0.001. Gender was dummy coded with 0.5 (male) and −0.5 (female).

**Table 2:** Regression results for the conditional indirect effect.

Regression Equation		Fit Index			Regression Coefficients	
Outcome Variables	Predictors	R	R^2^	F	β	t
Depression	Perceived family financial stress	0.33	0.11	19.59	0.07	4.76 ***
Suicidal ideation	Perceived family financial stress	0.72	0.52	94.10	0.01	0.92
	Depression				0.55	15.66 ***
	Parent attachment				−0.04	−3.33 ***
	Depression × Parent–child attachment				−0.13	−3.07 **

Note. ** *p* < 0.01; *** *p* < 0.001.

**Table 3:** Conditional indirect effects of parent attachment on suicidal ideation via depression based on bootstrapping technique.

Level of Parent Attachment	Indirect Effects	Boot SE	95% Bootstrap Lower Limit	95% Bootstrap Upper Limit
M − SD	0.05	0.01	0.03	0.07
M	0.04	0.01	0.02	0.06
M + SD	0.03	0.01	0.02	0.05

Note. M = Mean; SE = standard error.

## Data Availability

The data of this study are available from the corresponding author upon reasonable request.
